# Genetics of wild and mass-reared populations of a generalist aphid parasitoid and improvement of biological control

**DOI:** 10.1371/journal.pone.0249893

**Published:** 2021-04-13

**Authors:** Estelle Postic, Yannick Outreman, Stéphane Derocles, Caroline Granado, Anne Le Ralec

**Affiliations:** 1 IGEPP, INRAE, Institut Agro, Univ Rennes, Rennes, France; 2 AOPn Fraises de France, Estillac, France; 3 IGEPP, INRAE, Institut Agro, Univ Rennes, Le Rheu, France; Sichuan University, CHINA

## Abstract

Due to their ability to parasitize various insect species, generalist parasitoids are widely used as biological control agents. They can be mass-reared and released in agroecosystems to control several pest species in various crops. However, the existence of genetic differentiation among populations of generalist parasitoid species is increasingly recognized and this can be associated with an adaptation to local conditions or to a reduced range of host species. Moreover, constraints of mass-rearing conditions can alter genetic variation within parasitoid populations released. These features could be associated with a reduced efficiency of the control of targeted pest species. Here, we focused on strawberry greenhouses where the control of aphids with the generalist parasitoid *Aphidius ervi* appears to be inefficient. We investigated whether this inefficiency may have both genetic and ecological bases comparing wild and commercial populations of *A*. *ervi*. We used two complementary genetic approaches: one based on the mitochondrial marker COI and one based on microsatellite markers. COI analysis showed a genetic differentiation within the *A*. *ervi* species, but the structure was neither associated with the commercial/wild status nor with host species factors. On the other hand, using microsatellite markers, we showed a genetic differentiation between commercial and wild *A*. *ervi* populations associated with a loss of genetic diversity within the mass-reared populations. Our ecological genetics study may potentially explain the weak efficiency of biological control of aphids in protected strawberry crops and enable to provide some insights to improve biological control.

## Introduction

Due to their ability to switch among host species, generalist parasitoids are particularly interesting for inundative biological control [1] as they can potentially be mass-reared on a particular host and used to control several pest species. However, among natural populations of a parasitoid species, variations in the level of virulence on different host species may occur as a result of an adaptation to local conditions [2, 3]. Studies on intraspecific genetic structure of parasitoids have revealed that the degree of generalism of numerous species might be overestimated [4], as an increasing number of species previously assumed to be generalists have been found to comprise distinct populations or even cryptic species with a higher degree of specialization. Thus, when establishing a mass-reared population from a natural population, it is essential to determine its actual host range. Moreover, efficiency of control of the targeted pests implies adaptation to the environment of release, to the range of targeted species [5], but also to the defenses of the hosts [6]. All these adaptations rely on genetic diversity [7] and may be challenged by the constraints imposed on mass-reared populations of natural enemies. During this process, a genetic bottleneck may induce an initial loss of genetic diversity, increasing across generations by drift and/or inbreeding [8]. Lastly, mass-rearing of the agents often involves the use of a single host species and controlled climatic conditions for practical and economic reasons. These characteristics may impose selection for adaptation to mass-rearing conditions [9] and then a risk of maladaptation to the environment of release as well as to some targeted pest species.

The aphid parasitoid *Aphidius ervi* (Haliday) (Hymenoptera: Braconidae: Aphidiinae) is a generalist species described on more than twenty host species [10]. This hymenopteran is commercialized to control several aphid species like *Macrosiphum euphorbiae* (Thomas) or *Aulacorthum solani* (Kaltenbach) in greenhouse crops [11]. However, in some cases, failures of biological control of aphids with *A*. *ervi* have been reported [12]. This is the case of protected strawberry crops in France, where several potential aphid hosts of *A*. *ervi* coexist, the more frequent being *Acyrthosiphon malvae* (Mosley), *M*. *euphorbiae* and *Rhodobium porosum* (Sanderson) [13]. However, spontaneous colonization of greenhouses with *A*. *ervi* was observed on its three major aphid hosts on strawberry, suggesting that some wild populations of *A*. *ervi* would be more effective at controlling aphids in strawberry crops than mass-reared commercial ones [13].

Here, we investigated whether the inefficiency of inundative biological control in mass-reared populations of *A*. *ervi* may have a genetic basis, with two non-exclusive hypotheses. (1) There is a genetic differentiation between commercial and wild populations of *A*. *ervi* resulting from different levels of host specialization, the mass-reared parasitoids having a distinct and narrowest host range compared to wild ones. To test this hypothesis, we compared genetic diversity between mass-reared populations and wild populations from aphids on strawberries and on other cultivated and wild plants, using a fragment of the COI mitochondrial gene that allows to resolve high level of intraspecific differentiation. The association of such differences to host specialization has already been demonstrated in several species of Aphidiinae [14, 15]. We included samples coming from distant countries to check for a potential effect of geographical origin. (2) A lack of genetic diversity is present as a result of mass-rearing constraints. To test this hypothesis, we compared commercial and wild individuals of *A*. *ervi* genetically by using microsatellite markers to identify finer levels of differentiation. The wild individuals came from aphids on strawberries and on other crops, sampled at a large scale, in order to assess the effects of the aphid host plant, the aphid host species, and the geographic distance on genetic variability. Considering diverse wild putative populations, defined on the basis of their wild/commercial origin, their region and their aphid host, we investigated whether commercial and wild parasitoids differ in terms of both genetic structure and diversity.

## Materials and methods

### Biological material

We considered both ‘commercial’ and ‘wild’ *A*. *ervi* individuals. Commercial *A*. *ervi* were mass-reared individuals bought from three different suppliers in 2016, 2017 and 2019. Wild *A*. *ervi* individuals were specimens sampled in protected strawberry and cucumber crops and specimens from previous studies sampled on open fields. Table 1 details the number of *A*. *ervi* individuals from each origin and the number of individuals used for each analysis.

**Table 1:** *Aphidius ervi* samples used for genetic analyses.

Origin	Plant	Location / supplier	Aphid host	Number of samples used for COI analysis	Number of samples used for COI analysis (only confirmed *A*. *ervi*)	Number of samples used for microsatellite analysis	Source	COI Genebank accession number
Wild	*Fragaria × ananassa*	France—West	*Acyrthosiphon malvae*	14	14	10	This study	
Wild	*Fragaria × ananassa*	France—West	*Macrosiphum euphorbiae*	18	17	10	This study	
Wild	*Fragaria × ananassa*	France—West	*Rhodobium porosum*	42	36	16	This study	
Wild	*Fragaria × ananassa*	France—West	*Aulacorthum solani*	2	2	-	This study	
Wild	*Fragaria × ananassa*	France—West	*Aphis* sp.	1	1	-	This study	
Wild	*Fragaria × ananassa*	France—Center	*Acyrthosiphon malvae*	4	2	1	This study	
Wild	*Fragaria × ananassa*	France—Center	*Macrosiphum euphorbiae*	13	13	9	This study	
Wild	*Fragaria × ananassa*	France—Center	*Rhodobium porosum*	4	4	3	This study	
Wild	*Fragaria × ananassa*	France—Center	*Aulacorthum solani*	6	3	1	This study	
Wild	*Fragaria × ananassa*	France—East	*Macrosiphum euphorbiae*	1	1	1	This study	
Wild	*Fragaria × ananassa*	France—Southeast	*Acyrthosiphon malvae*	1	1	1	This study	
Wild	*Fragaria × ananassa*	France—Southeast	*Macrosiphum euphorbiae*	7	7	6	This study	
Wild	*Fragaria × ananassa*	France—Southeast	*Rhodobium porosum*	16	16	10	This study	
Wild	*Fragaria × ananassa*	France—Southwest	*Acyrthosiphon malvae*	18	16	37	This study	
Wild	*Fragaria × ananassa*	France—Southwest	*Macrosiphum euphorbiae*	34	33	25	This study	
Wild	*Fragaria × ananassa*	France—Southwest	*Rhodobium porosum*	29	28	24	This study	
Wild	*Cucumis sativus*	France—West	*Macrosiphum euphorbiae*	5	5	9	This study	
Wild	*Cucumis sativus*	France—West	*Aulacorthum solani*	3	3	4	This study	
Wild	*Cucumis sativus*	France—West	*Myzus persicae*	1	1	4	This study	
Wild	*Medicago sativa*	France—West	*Acyrthosiphon pisum*	4	4	126	COI: Derocles et al. 2016, Derocles et al. 2020; microsatellites: Zepeda-Paulo et al. 2016	KP983592, KP983593, KP983594,
								MW270086
Wild	*Medicago sativa*	France—East	*Acyrthosiphon pisum*	-	-	63	Zepeda-Paulo et al. 2016	
Wild	*Medicago sativa*	Algeria	*Acyrthosiphon pisum*	1	1	-	This study	
Wild	*Trifolium pratense*	France—West	*Acyrthosiphon pisum*	1	1	123	COI: Derocles et al. 2020; microsatellites: Zepeda-Paulo et al. 2016	MW270088
Wild	*Trifolium pratense*	France—East	*Acyrthosiphon pisum*	-	-	73	Zepeda-Paulo et al. 2016	
Wild	*Pisum sativum*	France—West	*Acyrthosiphon pisum*	4	4	-	This study	
Wild	*Pisum sativum*	UK	*Acyrthosiphon pisum*	3	3	-	Derocles et al. 2016	KP983602, KP983603, KP983604
Wild	*Vicia faba*	France—West	*Acyrthosiphon pisum*	6	6	-	This study	
Wild	*Triticum aestivum*	France—West	*Sitobion avenae*	3	3	-	Derocles et al. 2016	KP983599, KP983600, KP983601
Wild	*Triticum aestivum*	France—East	*Sitobion avenae*	2	2	-	Derocles et al. 2016	KP983597, KP983598
Wild	*Triticum aestivum*	UK	*Sitobion avenae*	3	3	-	Derocles et al. 2016	KP983605, KP983606, KP983607
Wild	*× Triticosecale*	France—West	*Sitobion avenae*	6	6	-	This study	
Wild	*Solanum tuberosum*	Algeria	*Myzus persicae*	6	6	-	This study	
Wild	*Solanum tuberosum*	Algeria	*Macrosiphum euphorbiae*	1	1	-	This study	
Wild	*Sonchus* sp.	UK	*Hyperomyzus lactucae*	3	3	-	Derocles et al. 2016	KP983608, KP983609, KP983610
Wild	*Anthriscus sylvestris*	France—West	*Cavariella aegopodii*	1	1	-	Derocles et al. 2020	MW270090
Wild	*Brassica rapa*	France—West	*Myzus persicae*	1	1	-	Derocles et al. 2020	MW270091
Wild	*Picris hieracioides*	France—West	*Hyperomyzus picridis*	1	1	-	Derocles et al. 2020	MW270089
Wild	*Urtica dioica*	France—West	*Microlophium carnosum*	1	1	-	Derocles et al. 2020	
Commercial	Unknown	Supplier 1	Unknown	19	19	60	This study	
Commercial	Unknown	Supplier 2	Unknown	7	7	8	This study	
Commercial	Unknown	Supplier 3	Unknown	7	7	13	This study	
TOTAL				299	283	637		

#### *Aphidius ervi* sampling in protected crops

We sampled aphid mummies (*i*.*e*. dead aphids containing an immature parasitoid) in strawberry greenhouses in 2017 and 2018, in five regions of France, using the sampling design described in [13]. For both years, protected strawberry crops were sampled during the spring (from April to May) and the summer (from August to September). Six to 13 greenhouses were sampled within each region and in each season of a given year. Once in a greenhouse, we randomly selected between 25 to 30 sampling locations distributed throughout the monitored greenhouse. A sampling location consisted of a portion of a crop row about 2 meters long that was examined for the presence of aphid mummies. Using the same sampling procedure, in 2019, we collected mummies in 14 additional strawberry greenhouses in ‘Southwest’ and in six cucumber (*Cucumis sativus*) greenhouses in ‘West’ region. The collected mummies were placed individually into 1.5 mL plastic tubes waiting for parasitoids emergence in the laboratory. After emergence, adult parasitoids were placed in 96% ethanol and the parasitoid species was identified using morphological criteria before DNA extraction [16]. The specific diversity of emerged parasitoids was described in [13], in the present study only parasitoids identified as *Aphidius ervi* were considered. Their aphid hosts were also identified based on morphological traits of the mummy [17].

#### Additional ‘wild’ *A*. *ervi* specimens

In addition to the sampling of wild *A*. *ervi* individuals in protected crops, we considered in our analyses *A*. *ervi* individuals sampled in various agroecosystems in France, Algeria, and the United Kingdom. This included data acquired in the present study and in previously published studies (Table 1). The latter were *A*. *ervi* individuals collected on Vicia faba, Medicago sativa, Pisum sativum, × Triticosecale, and Solanum tuberosum fields in France and Algeria in 2017

### Analysis of mitochondrial DNA data

#### COI amplification and sequencing

Parasitoid DNA was extracted using the QIAGEN DNeasy kit following the manufacturer’s protocol for individuals collected in 2017 (non-destructive extraction) and a salting-out protocol [18] for individuals collected in 2018 (destructive extraction). The barcoding region of the mitochondrial COI gene was amplified using the primers LCO1490 and HCO2198 [19]. PCR amplifications were carried out following [20]. PCR products were purified and Sanger sequenced by Genoscreen (Lille, 59, France).

#### Phylogenetic placement

The 615-bp sequences were edited using Geneious Prime 2019.2.1 and aligned with MUSCLE v. 3.8 [21]. Alignments were translated into amino acids to detect frameshifts or stop codons indicating pseudogenes [22]. To verify the identity of sampled *A*. *ervi* individuals, a phylogenetic placement was performed using 450 sequences of Aphidiinae from Genebank and BOLD (S1 Table for details of sequences), including 255 sequences of *A*. *ervi* to take into account the intraspecific genetic variability. Phylogeny was constructed using Bayesian inference (hereafter noted BI) using MrBayes 3.2.6 [23] (Chain length: 10^6^, burn-in length: 10^5^) and by Maximum Likelihood (hereafter noted ML) using PhyML 3.0 with 1000 bootstrap replicates [24]. Belonging to another subfamily of Braconidae (Microgastrinae), *Cotesia flavipes* was used as an outgroup [25]. The best evolutionary model of sequences (GTR + I + G) was determined using jModelTest [26] with the Akaike information criteria corrected for small sample sizes [27]. Individuals identified as a species different from *A*. *ervi* by their position on the tree, were discarded from further analyses.

#### COI variability in *A*. *ervi*

To detect putative clades within *A*. *ervi* individuals sampled in strawberry greenhouses and other agroecosystems, both ML and BI trees were built with *Diaeretiella rapae* (Hymenoptera: Aphidiinae) (GenBank: JN620613.1) as outgroup. The best evolutionary model of sequences (HKY + I) was determined using jModelTest with the Akaike information criteria corrected for small sample sizes. To represent the genetic diversity of COI in *A*. *ervi*, a Median Joining haplotype network [28] was constructed using PopART v.1.7 [29].

### Microsatellite analyses

#### Microsatellite amplification

*Aphidius ervi* is a haplodiploid species (*i*.*e*. diploid females origin from fertilized eggs while haploid males develop from unfertilized eggs), so only females were used for genotyping. Seven loci developed by Zepeda-Paulo et al. [30] were amplified: Ae01, Ae03, Ae06, Ae16, Ae27, Ae29 and Ae33. DNA was extracted using the same protocol as the mtDNA amplification one (details of samples in Table 1). Each PCR reaction was set-up in 10 μL reaction volume containing 1 μL of genomic DNA, 0.5 X of buffer, 1.25 mM MgCl2, 0.2 mM of dNTPs, 0.25 μM of reverse primer, 0.25 μM of forward primer tailed with M13, 0.25 μM of M13 fluorescent labelled primer, and 0.25 U of Taq polymerase. The PCR were conducted using the following cycling conditions: initial denaturation at 94°C for 5 minutes, first cycle of DNA amplification (repeated 20 times) with a denaturation step at 94°C for 20 seconds, hybridization at 55°C for 20 s, elongation at 72°C for 30 s; second cycle of M13 amplification with 20 repetitions of the following steps: 94°C for 20 s, 53°C for 20 s and 72°C for 30 s. The PCR ends with a final elongation at 72°C for 5 min. Allele sizes were identified using the automatic calling and binning procedure of GENEMAPPER v.4.1 (Applied Biosystems) and were confirmed manually.

#### Definition of putative populations of parasitoids

For microsatellite data analyses, different putative populations and subpopulations of *A*. *ervi* individuals were considered. First, we distinguished ‘commercial’ individuals from all ‘wild’ ones. Secondly, the ‘wild’ individuals were split into four putative subpopulations according to the crop where they were sampled: (1) ‘*Cucumis*’ (cucumber), (2) ‘*Fragaria*’ (strawberry), (3) ‘*Medicago*’ (alfalfa), and (4) ‘*Trifolium*’ (clover). Finally, we considered individuals sampled in strawberry greenhouses (*i*.*e*. ‘*Fragaria*’) and split them according to the sampled region (*i*.*e*. ‘West’, ‘Center’, ‘Southeast’, or ‘Southwest’–‘East’ was excluded as only one individual was collected there) or to the aphid host species (*i*.*e*. ‘*A*. *malvae’*, ‘*M*. *euphorbiae’* or ‘*R*. *porosum’*).

#### Genetic diversity

For all putative populations and subpopulations, we estimated the average observed (Ho) and expected (He) heterozygosity using GenAlEx (v. 6.5) [31]. The average inbreeding coefficient (F_IS_), and the standardized allelic richness (using a resampling method) (Ar) were calculated with the R package ‘*diveRsity*’ [32, 33]. Confidence intervals of Ar and F_IS_ were estimated using 1000 bootstrap replicates.

#### Population structure

The differentiation between each pair of putative populations and subpopulations was estimated using F_ST_ and Jost’s D with the R package ‘*diveRsity*’ [32]. While F_ST_ can be biased downward for loci with many alleles, Jost’s D [34] is best suited for describing the allelic differentiation among populations [35]. Bias corrected confidence intervals (95%) were calculated using 1000 bootstrap replicates.

To explore population structure without *a priori* on the defined putative populations, we used the Bayesian clustering algorithm implemented in STRUCTURE v. 2.3.4 [36] on the whole dataset. We used the admixture model with correlated allele frequencies. Ten runs were performed per value of K (number of clusters) from 1 to 15. For each run, a burn-in period of 100 000 iterations followed by 100 000 Markov chain Monte Carlo (MCMC) repetitions were applied. The most probable value of K was estimated using the Evanno statistic ΔK [37] implemented in Structure Harvester v0.6.94 [38]. To account for variability among runs, the coefficient of ancestry *Q* was averaged for each individual on all the runs performed. As the reliable detection of population structure might be affected by uneven sampling [39], we also ran STRUCTURE on the subsamples composed of ‘wild’ *A*. *ervi* and composed of parasitoids from *Fragaria* with the same parameters as on the whole dataset with values of K from 1 to 10.

Additionally, to visualize the structure of the whole dataset and the ‘wild’ and ‘*Fragaria*’ subsamples, we used a Discriminant Analysis of Principal Components (DAPC) [40]. DAPC was performed with the R package ‘*adegenet*’ [41], using cross-validation to define the best number of principal components and discriminant functions.

#### Parasitoid origin inference

In some sampled strawberry and cucumber greenhouses, releases of commercial parasitoids were done by producers before our sampling. To detect putative commercial parasitoids among the ‘wild’ parasitoids sampled in greenhouses of strawberries and cucumber, we performed an assignment test using GENECLASS2 [42] with two defined putative populations: ‘commercial’ and ‘wild’. We used the L_home_/L_max_ likelihood computation, where L_home_ is the likelihood of an individual being assigned to the population from which it was sampled and L_max_ is the maximum likelihood for all populations considered. We used the Bayesian method of Rannala and Mountain [43] with an alpha of 0.05 and 10 000 repetitions of MCMC re-sampling algorithm [44].

## Results

### Phylogenetic analyses and haplotype networks

**Fig 1:**
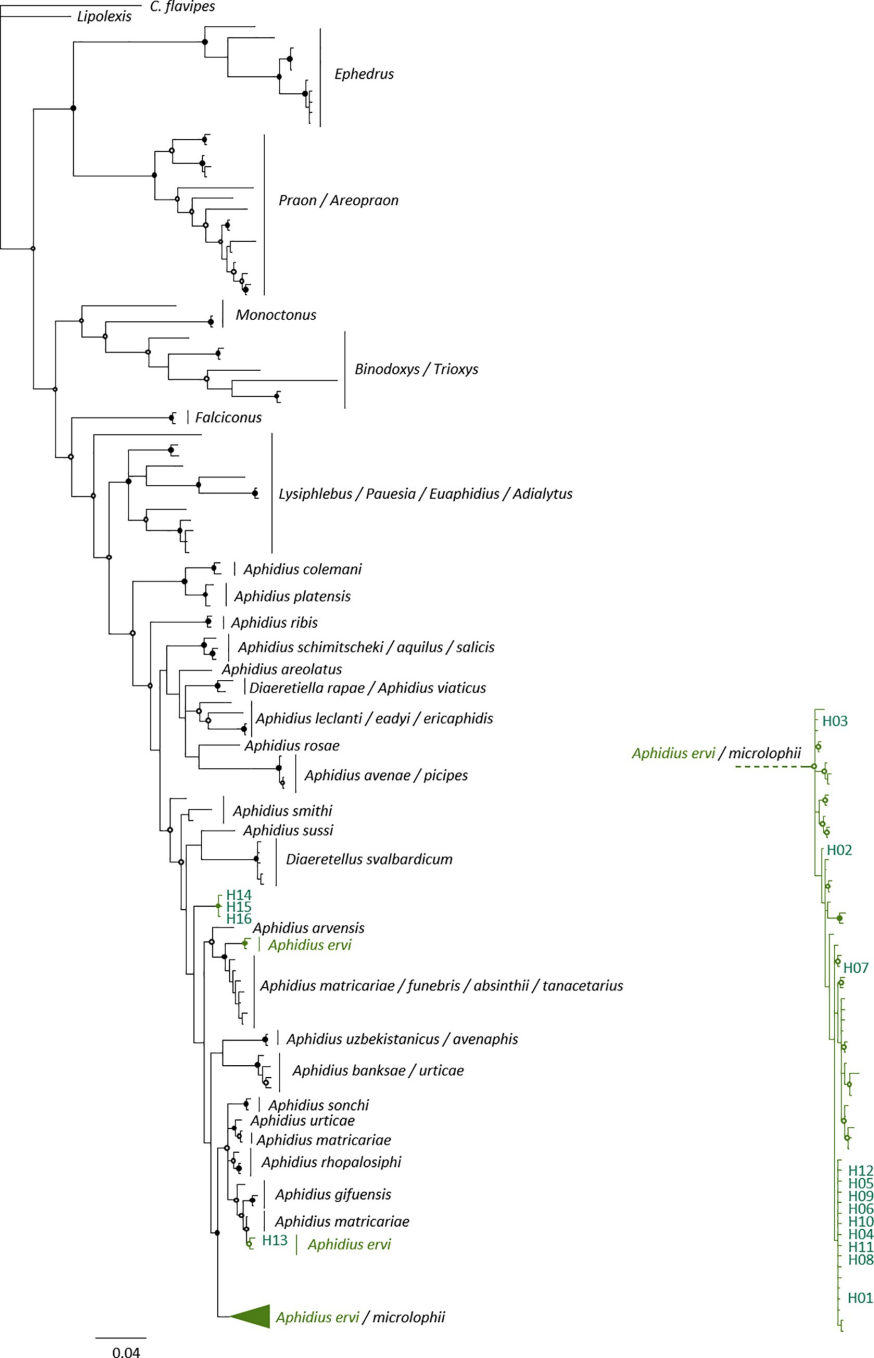
Phylogenetic tree of Aphidiinae reconstruction using Bayesian Inference (BI) and Maximum Likelihood (ML). Solid circles show nodes supported by both BI and ML analyses (posterior probability > 0.9 and bootstrap value > 90). Open circles show nodes supported by BI analysis only. Individuals identified as *Aphidius ervi* using morphological characters are colored in green. The tree was reconstructed using sequences from BOLD, GenBank and from the present study. Haplotypes sampled in this study are numbered from H01 to H16. Scaling is expressed in the proportion of substituted bases per site.

Most of *A*. *ervi* COI sequences from BOLD / GenBank formed a single cluster in the BI and ML trees (**Fig 1**). Within this main cluster, BI tree (and ML tree to a lesser extent) identifies clades with a posterior probability of 0.9. Three haplotypes (15, 4 and 1 individuals respectively, in green on the figure) from databases were not grouped inside the main cluster. Parasitoids identified morphologically as *A*. *ervi* belonged to 16 COI haplotypes. Four haplotypes (H13, H14, H15 and H16 on **Fig 1**), including 16 individuals from strawberry greenhouses, were highly different from others and were not grouped in the main cluster of *A*. *ervi* sequences from databases. H13 grouped with another sequence of *A*. *ervi* from databases within a cluster composed of *Aphidius gifuensis* and *Aphidius matricariae*. H14, H15 and H16 formed a cluster differentiated from all other *Aphidius* species. As their assignment to the *A*. *ervi* species was unsure, these four haplotypes were not included in the following analyses.

**Fig 2:**
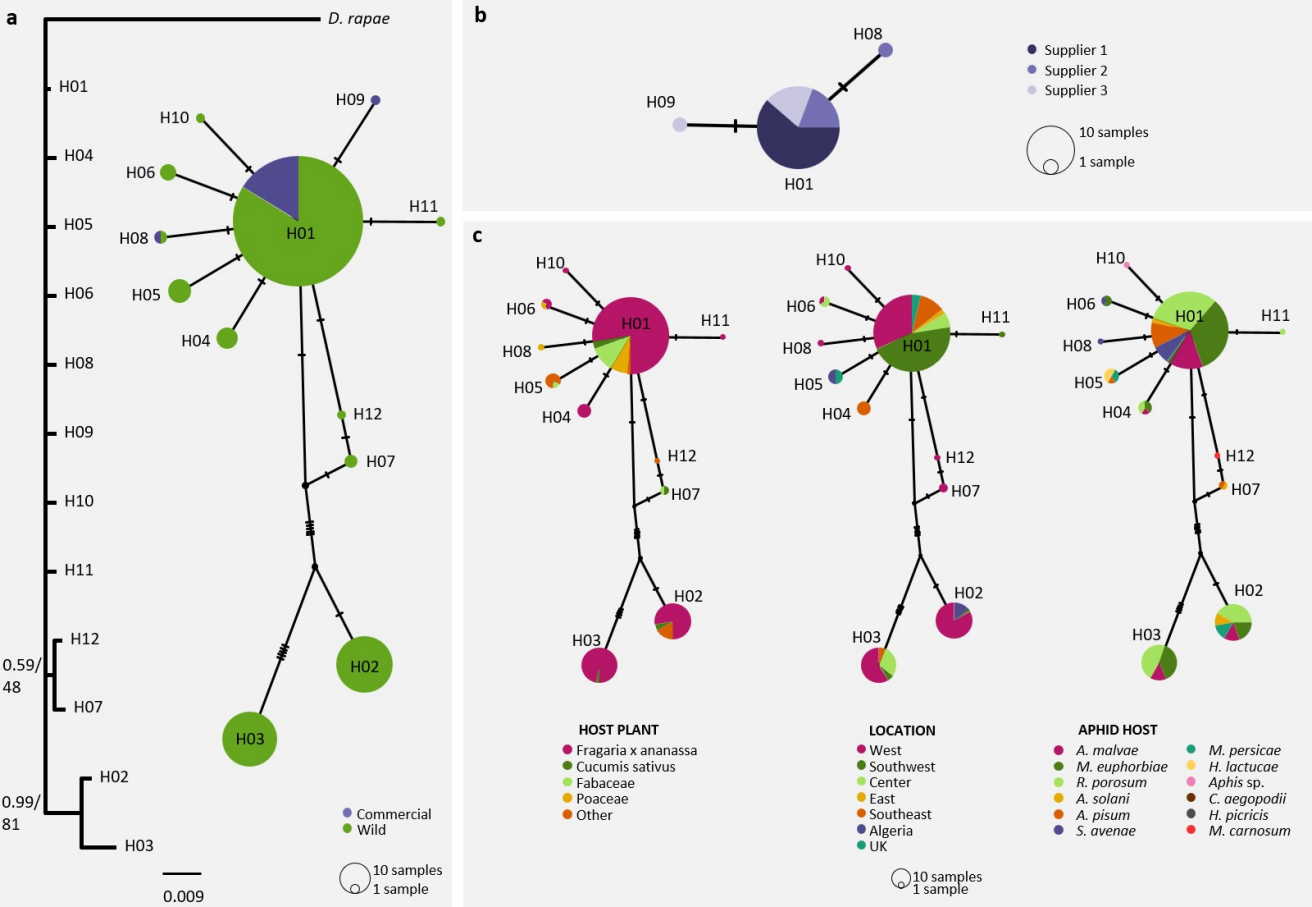
Genetic diversity of *Aphidius ervi* based on COI sequences. **A**: Bayesian inference (BI) / Maximum Likelihood (ML) tree and Median Joining haplotype network of commercial and ‘wild’ parasitoids sampled in strawberry greenhouses and other agroecosystems. On the tree, the first value at nodes shows posterior probability (BI analysis) and the second value shows bootstrap value (ML analysis). Scaling is expressed in the proportion of substituted bases per site. **B**: Median Joining network of commercial parasitoids according to the supplier. **C**: Median Joining network of ‘wild’ parasitoids according to the host plant, the location and the aphid host. In haplotype networks, hatch marks indicate a mutation between haplotypes.

Among the 12 remaining haplotypes (283 individuals, Table 1), 67% of the individuals belonged to the haplotype H01, 8% belonged to closely related haplotypes (H04 to H12) and 25% belonged to the most differentiated group of haplotypes composed of H02 and H03 (posterior probability > 0.9; **Fig 2A**). Commercial parasitoids were not structured strictly by the supplier. About 94% of commercial *A*. *ervi* belonged to the main haplotype H01 (along with 64% of the ‘wild’ parasitoids) and only two individuals belonged to the haplotypes H08 and H09 (**Fig 2B**). ‘Wild’ *A*. *ervi* belonged to 11 haplotypes. No clear pattern of mtDNA differentiation was associated with host plants. However, neither individuals from Fabaceae (*Medicago sativa*, *Trifolium pratense*, *Vicia faba* and *Pisum sativum*) nor from Poaceae (× *Triticosecale* and *Triticum aestivum*) belonged to the most differentiated haplotypes (H02 and H03; **Fig 2C**). We found neither geographic differentiation, independently of the geographical scale considered nor genetic pattern associated with the aphid host species on haplotype networks (**Fig 2C**).

### Genetic diversity within parasitoid putative populations

Observed heterozygosity (*Ho*) ranged from 0.39 to 0.69 in the different putative populations (Table 2). *Ho* was lower in ‘commercial’ parasitoids (0.44) compared to ‘wild’ ones (0.50) and slightly lower compared to ‘wild’ parasitoids sampled in strawberry greenhouses (0.47). In both cases, standard errors of *Ho* overlapped between commercial and wild parasitoids. In all cases, *Ho* was lower than unbiased expected heterozygosity (*uHe*). Standardized allelic richness (*Ar*) was much lower in ‘commercial’ *A*. *ervi* (4.4) compared to ‘wild’ parasitoids (10.2) (Table 2). This was also the case when ‘commercial’ parasitoids were compared to the subset of ‘wild’ parasitoids sampled in strawberry greenhouses (*Ar* = 8.4). However, no inbreeding was detected in the ‘commercial’ population (F_IS_ = 0.14) contrary to the ‘wild’ populations (F_IS_ = 0.30) (Table 2). Among the ‘wild’ putative subpopulations related to the aphid host plant, *Ar* was similar (from 5.2 to 6.4). Inbreeding was detected in all the putative subpopulations except the ‘*Cucumis*’ one (Table 2). Among the putative subpopulations from strawberry greenhouses defined according to the region, *Ar* was slightly lower in ‘Southeast’ (3.6) and ‘Center’ (4.2) compared to ‘Southwest’ (5.0) and ‘West’ (5.2). Inbreeding was detected in all the subpopulations except the ‘Southwest’ one (Table 2). Among the ‘*Fragaria*’ putative subpopulations defined according to the aphid host species of the parasitoid individual, *Ar* was similar (from 6.6 to 7.1).

**Table 2:** Genetic variability of *Aphidius ervi* on seven microsatellite loci in each putative population and subpopulation.

Putative population	Number of individuals	Allelic richness (CI)	*uHe* (SE)	*Ho* (SE)	F_IS_(CI)
**All *A*. *ervi***					
Wild	556	10.2	0.72	0.50	0.30
		9.3–11.1	(0.04)	(0.03)	0.27–0.34
Wild—*Fragaria*	154	8.4	0.64	0.47	0.26
		7.6–9.3	(0.07)	(0.04)	0.21–0.31
Commercial	81	4.4	0.51	0.44	0.14
		4.0–4.7	(0.06)	(0.05)	0.06–0.20
**’wild’**					
*Cucumis*	17	5.2	0.70	0.63	0.08
		4.6–5.7	(0.05)	(0.06)	-0.09–0.18
*Fragaria*	154	5.7	0.65	0.47	0.26
		4.9–6.7	(0.07)	(0.04)	0.21–0.31
*Medicago*	189	6.3	0.72	0.51	0.30
		5.4–7.3	(0.03)	(0.02)	0.24–0.36
*Trifolium*	196	6.4	0.73	0.51	0.29
		5.4–7.3	(0.04)	(0.02)	0.23–0.35
**’*Fragaria*’—region**					
Southeast	17	3.6	0.52	0.39	0.14
		3.0–3.9	(0.1)	(0.07)	-0.04–0.27
Southwest	86	5.0	0.60	0.45	0.23
		4–5.9	(0.08)	(0.05)	0.15–0.30
West	36	5.2	0.70	0.52	0.23
		4.4–6.0	(0.05)	(0.04)	0.10–0.34
Center	14	4.2	0.66	0.51	0.24
		3.6–4.6	(0.05)	(0.09)	-0.02–0.40
**’*Fragaria*’—aphid host**					
*R*. *porosum*	53	6.6	0.64	0.48	0.23
		6.0–7.1	(0.07)	(0.05)	0.13–0.30
*M*. *euphorbiae*	51	6.7	0.64	0.50	0.21
		6.0–7.1	(0.07)	(0.06)	0.10–0.29
*A*. *malvae*	49	7.1	0.65	0.42	0.33
		6.4–7.9	(0.06)	(0.03)	0.20–0.42

Allelic richness is standardized according to the smallest sample size within each population considered. *uHe*: unbiased expected heterozygosity. *Ho*: observed heterozygosity. F_IS_: fixation index. CI: confidence interval. SE: standard error.

### Genetic differentiation among parasitoid putative populations

**Fig 3:**
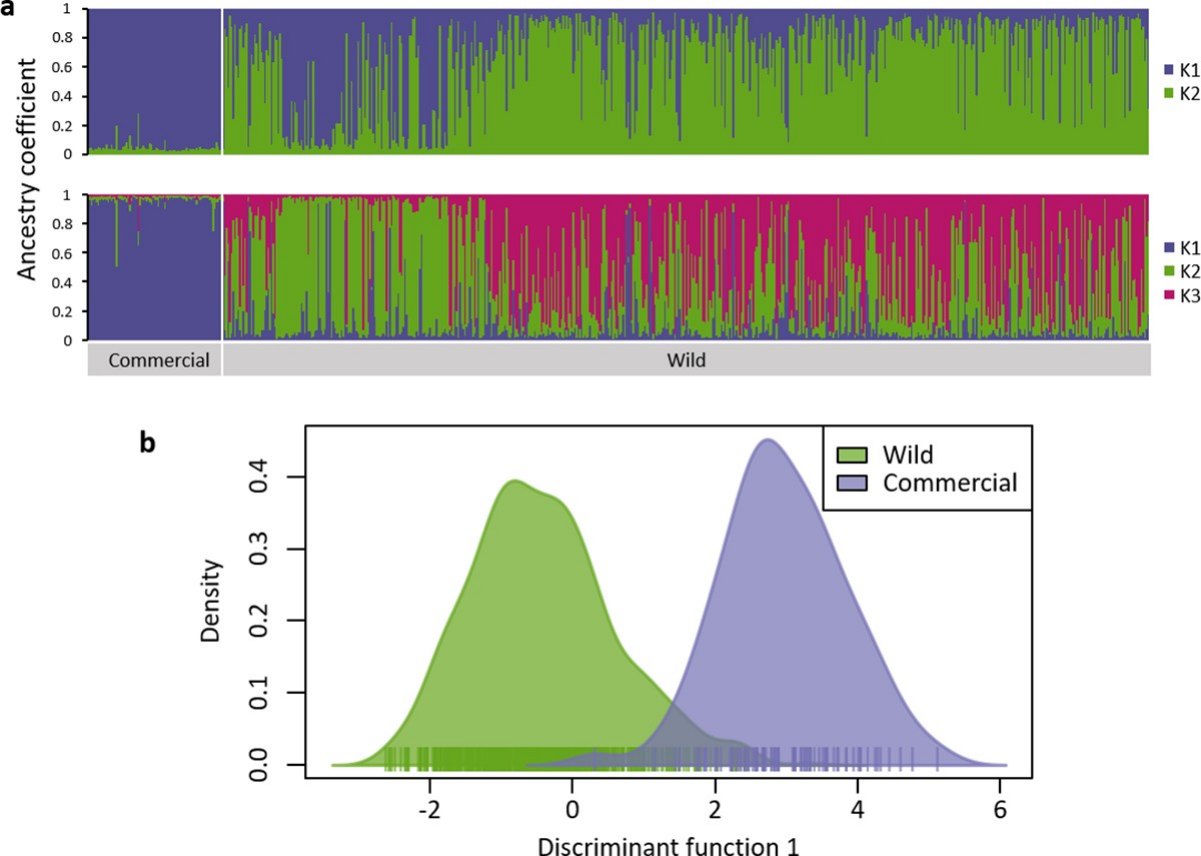
Genetic structure of *Aphidius ervi* based on seven microsatellite loci for all parasitoids (commercial and wild): (a) inference of population structure using Bayesian clustering with the program STRUCTURE (K = 2 and K = 3, the most probable numbers of clusters are presented, coefficient of ancestry is an average on ten runs) and (b) Discriminant Analysis of Principal Components according to the origin of the parasitoids (wild or commercial).

Considering the whole dataset, differentiation indices between the ‘commercial’ and the ‘wild’ populations were high (F_ST_ = 0.118; D = 0.173; Table 3). Using STRUCTURE, the most likely numbers of clusters were 2 (ΔK = 12.1) and 3 (ΔK = 9.5, S1A Fig) so results for both numbers of clusters are presented (**Fig 3A**). With a threshold of coefficient of ancestry *Q* > 0.8, most ‘commercial’ individuals were assigned to K1 (98% with K = 2, 95% with K = 3). On the other hand, ‘wild’ parasitoids were mostly assigned to other clusters or undetermined (49% to K2 and 37% undetermined with K = 2; 22% to K2, 24% to K3 and 51% undetermined with K = 3). Using DAPC, 97% of individuals were assigned to their putative population of origin (‘wild’: 98% and commercial: 93%; **Fig 3B**).

**Table 3:** Genetic differentiation between putative populations and subpopulations of *Aphidius ervi*: Pairwise F_ST_ and Jost’s D values based on seven microsatellite loci with Confidence Intervals (CI).

Comparison	F_ST_ (CI)	Jost’s D (CI)
**Commercial vs Wild**	**0.118**	**0.173**
** **	0.102–0.135	0.145–0.206
**’wild’**		
***Cucumis* vs *Fragaria***	**0.045**	**0.053**
***Cucumis* vs *Medicago***	0.017–0.077	0.009–0.111
	**0.031**	0.047
***Cucumis* vs *Trifolium***	0.008–0.058	- 0.003–0.115
	**0.026**	**0.050**
***Fragaria* vs *Medicago***	0.004–0.053	0.000–0.115
	**0.062**	**0.077**
***Fragaria* vs *Trifolium***	0.045–0.073	0. 057–0.101
	**0.059**	**0.075**
***Medicago* vs *Trifolium***	0.045–0.073	0.057–0.098
	0.000	0.001
** **	-0.003–0.004	-0.005–0.009
**’*Fragaria*’–region**		
**Southeast vs Southwest**	**0.057**	0.030
**Southeast vs West**	0.025–0.098	-0.004–0.080
	**0.083**	**0.093**
**Southeast vs Center**	0.045–0.128	0.037–0.170
	**0.099**	**0.091**
**Southwest vs West**	0.040–0.171	0.017–0.194
	**0.054**	**0.089**
**Southwest vs Center**	0.031–0.083	0.044–0.147
	**0.069**	**0.094**
**West vs Center**	0.039–0.110	0.054–0.145
	0.016	0.015
** **	-0.010–0.061	-0.030–0.089
***’Fragaria’*—aphid host**		
***R*. *porosum* vs *M*. *euphorbiae***	**0.014**	**0.027**
***R*. *porosum* vs *A*. *malvae***	0.000–0.033	0.004–0.055
	0.012	0.011
***M*. *euphorbiae* vs *A*. *malvae***	-0.003–0.032	-0.009–0.037
	0.013	0.012
** **	-0.002–0.034	-0.006–0.042

**Fig 4:**
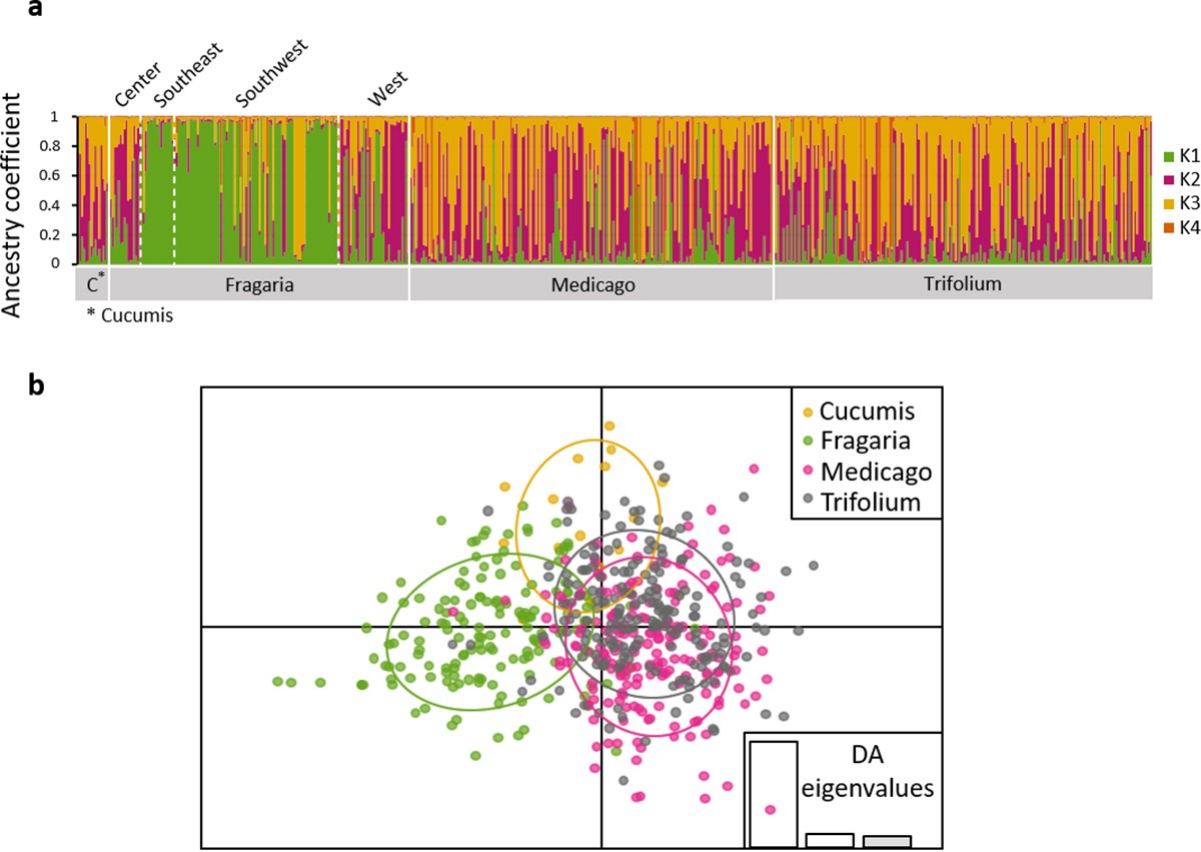
Genetic structure of wild *Aphidius ervi* based on seven microsatellite loci: (a) inference of population structure using Bayesian clustering with the program STRUCTURE (K = 4, the most probable number of clusters is presented, coefficient of ancestry is an average on ten runs) and (b) Discriminant Analysis of Principal Components according to the host plant on which individuals were collected. Putative populations are distinguished by colors and inertia ellipses.

Considering the ‘wild’ parasitoid individuals sampled on four different plants, differentiation indices were low (F_ST_: from 0 to 0.062, D: from 0.001 to 0.077) but significantly different from zero except between the ‘*Medicago*’ and the ‘*Trifolium*’ putative subpopulations. The highest differentiation was observed for ‘*Fragaria*’ compared to both ‘*Medicago*’ and ‘*Trifolium*’ (Table 3). Using STRUCTURE, the optimal number of clusters was 4 (ΔK_wild_ = 23.8, S1B Fig) followed by 3 (ΔK_wild_ = 15.6) and 2 (ΔK_wild_ = 12.6), then we present results for K_wild_ = 4 (see S2 Fig for K_wild_ = 2 and K_wild_ = 3). Parasitoids from the ‘*Fragaria*’ putative subpopulation were mainly assigned to the cluster K1_wild_ (51%; **Fig 4A**), this was mostly due to individuals from ‘Southeast’ and ‘Southwest’ (**Fig 6A**). In ‘*Cucumis*’, ‘*Medicago*’ and ‘*Trifolium*’ putative subpopulations most individuals were not assigned to any cluster (from 48 to 71%; **Figs 4A and 6A**). DAPC confirmed that the main source of structure occurred between ‘*Fragaria*’ and other putative subpopulations (**Fig 4B**), 82% of individuals from ‘*Fragaria*’ were assigned to their putative population of origin against 53% for ‘*Cucumis*’, 61% for ‘*Medicago*’ and 62% for ‘*Trifolium*’.

**Fig 5:**
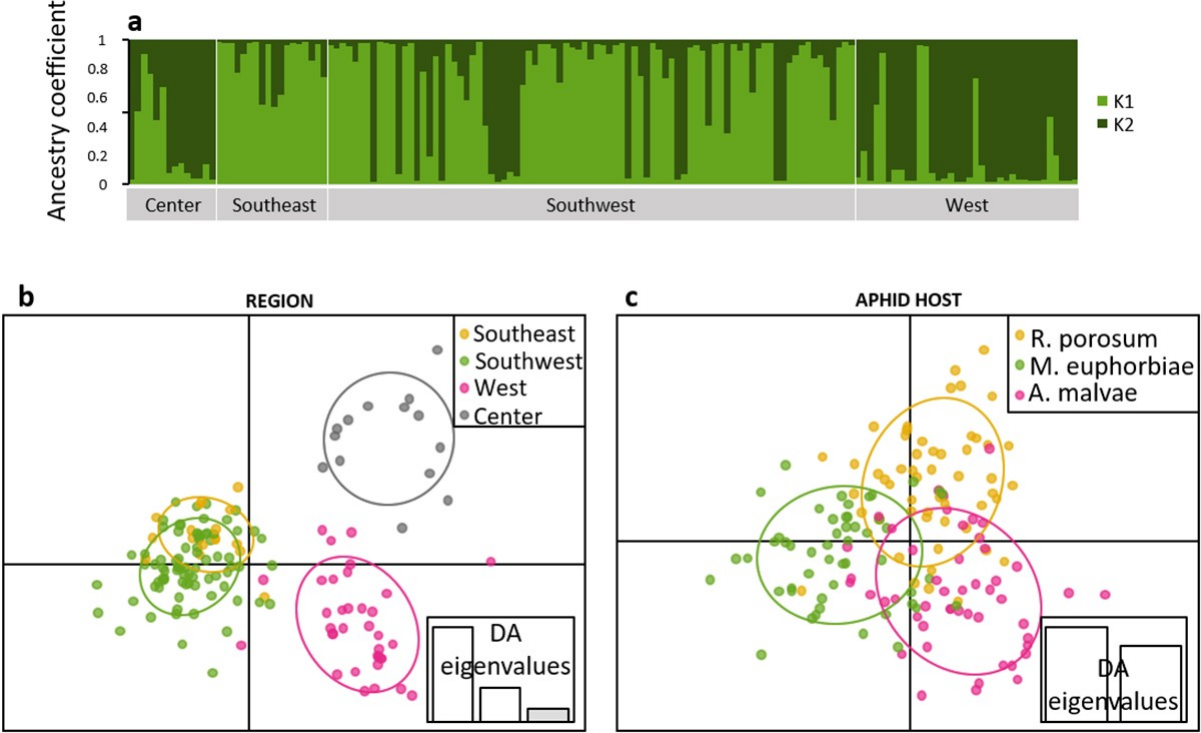
Genetic structure of wild *Aphidius ervi* collected in strawberry greenhouses based on seven microsatellite loci: (a) inference of population structure using Bayesian clustering with the program STRUCTURE (K = 2, the most probable number of clusters is presented, coefficient of ancestry is an average on ten runs) and Discriminant Analysis of Principal Components according to (b) the region of sampling and (c) to the aphid host. Putative populations are distinguished by colors and inertia ellipses.

Among pairs of locations within ‘*Fragaria*’, F_ST_ and Jost’s D values were significantly different from zero except between ‘West’ and ‘Center’ and between ‘Southeast’ and ‘Southwest’ (Table 3). The optimal number of clusters was 2 (ΔK_*Fragaria*_ = 48.7, S1C Fig). Individuals from the two southern regions of France ‘Southeast’ and ‘Southwest’ were mostly assigned to the cluster K1_*Fragaria*_ (‘Southeast’: 76%, ‘Southwest’: 66%). Conversely, individuals from the two northern regions ‘Center’ and ‘West’ were mostly assigned to the cluster K2_*Fragaria*_ (‘Center’: 64%, ‘West’: 81%; **Fig 5A**). DAPC also showed a differentiation between northern and southern regions (**Fig 5B**). The percentages of assignment to the region of origin were 88% for ‘Southeast’, 97% for ‘Southwest’, 94% for ‘West’ and 93% for ‘Center’.

**Fig 6:**
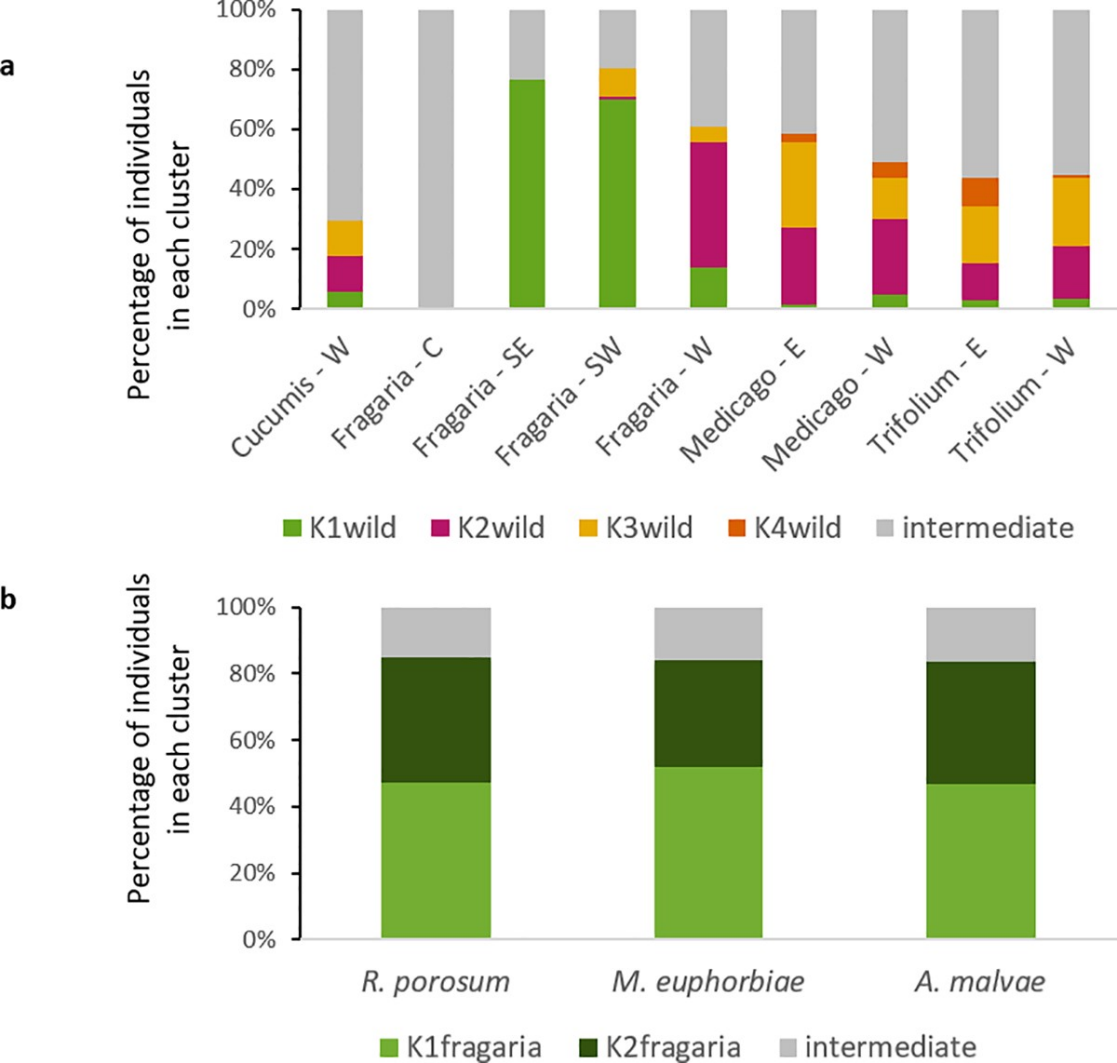
Percentage of individuals assigned in each cluster (with a threshold of coefficient of ancestry *Q* > 0.8) using Bayesian clustering with the program STRUCTURE. (a) ‘wild’ individuals according to the host plant and the region of sampling with four clusters. (b) ‘wild’ individuals from strawberry greenhouses according to the aphid host with two clusters. W: ‘West’; SW: ‘Southwest’; C: ‘Center’; E: ‘East’; SE: ‘Southeast’.

Finally, among pairs of aphid hosts within ‘*Fragaria*’, differentiation was very low (F_ST_: from 0.012 to 0.014, D: from 0.011 to 0.027) and significantly different from zero only between ‘*R*. *porosum*’ and ‘*M*. *euphorbiae*’. A similar proportion of individuals emerging from each species was assigned to K1_*Fragaria*_ (47 to 52%) and to K2_*Fragaria*_ (32 to 38%; **Fig 6B**). DAPC showed stronger differentiation between aphid species than STRUCTURE (**Fig 5C**), 75% of individuals from ‘*R*. *porosum*’ were assigned to the correct cluster, 78% for ‘*M*. *euphorbiae*’ putative population and 71% for ‘*A*. *malvae*’ putative population.

### Inference of the parasitoid origin in greenhouses

Among the 171 parasitoids sampled in strawberry and cucumber greenhouses, 41 individuals were sampled in greenhouses where commercial *A*. *ervi* had been released. Using assignment with GENECLASS2, one individual from the ‘commercial’ putative population was considered as ‘wild’ and 13 individuals from greenhouses were considered as ‘commercial’. Among these 13 parasitoids, 7 came from greenhouses with a commercial release.

## Discussion

Studying genetics of mass-reared and wild parasitoid populations may have implications for biological control, explaining its failure in some contexts. Here, we identified some genetic differentiation in aphid parasitoid individuals using the mitochondrial marker COI, but factors leading to this genetic structure have not been identified (*i*.*e*. neither the commercial/wild status, the geography, the aphid host species nor the crop plant), confirming the generalist status of *A*. *ervi*. On the other hand, using microsatellite markers, we showed a differentiation between ‘commercial’ and ‘wild’ *A*. *ervi* that may potentially explain the weak efficiency of biological control of aphids in protected strawberry crops. The hypothesis of a loss of genetic diversity in mass-reared populations leading to a low adaptation ability of the commercial *A*. *ervi* to control aphids on strawberries appeared the most likely, but further research would be needed to explore the phenotypical outcomes of such genetic diversity loss. From these results, we provide some insights to improve biological control with aphid parasitoids in greenhouse crops.

### Patterns of genetic variation identified with mitochondrial DNA

COI gene is widely used to infer the phylogeny of species [45] or to explore intraspecific genetic variability, including identifying cryptic species [4]. The trees reconstructed from sequences from databases and from our own study revealed that some individuals identified as *A*. *ervi* were placed in a clade separate from the bulk of the other *A*. *ervi* specimens. This could result from misidentification, but the rugose aspect of the anterolateral area of the petiole in *A*. *ervi* makes its identification quite easy [16]. Distant sequences could also result from the presence of COI copies in the nuclear genome of *A*. *ervi* (nuclear mitochondrial pseudogenes) [46] but the absence of both stop codons and sequence ambiguities makes this hypothesis unlikely [47]. An alternative hypothesis is the existence of cryptic species, hidden by a low level of morphological diversification [48]. Unruh et al. [49] already observed that *A*. *ervi* seemed to be a complex of species, although this differentiation was observed among geographically distant populations (Europe and the Mediterranean region / Japan / Pakistan).

Besides, considering only individuals belonging to the *A*. *ervi* clade, mtDNA analysis revealed low genetic variation for most individuals. Indeed, most of the parasitoids from strawberry crops and commercial insectaries but also from diverse habitats belonged to a single haplotype and nine very closely related haplotypes. However, we also identified two differentiated haplotypes that were not associated with the region, the aphid host plant, the aphid host species or the commercial/wild status. In a different portion of mtDNA (a portion of COI and COII), Hufbauer et al. [50] also found differentiated haplotypes of *A*. *ervi* and hypothesized the existence of subspecies or different species in *A*. *ervi* without any link to a particular host. The lack of genetic differentiation correlated with aphid host species confirms the generalism of *A*. *ervi*, which seems to be uncommon within Aphidiinae [14, 51].

### Patterns of genetic variation and diversity identified with microsatellite markers

As already observed with other parasitoid species [52], we showed a strong differentiation between ‘commercial’ and ‘wild’ *A*. *ervi*. Moreover, a difference in genetic diversity was found: allelic richness was reduced in ‘commercial’ parasitoids compared to the ‘wild’ ones. However, observed heterozygosity was not significantly different between ‘commercial’ and ‘wild’ parasitoids. When founding a mass-reared population, allelic richness is more sensitive to bottlenecks compared to heterozygosity while heterozygosity is highly sensitive to inbreeding [53]. Both the loss of alleles and the absence of detection of inbreeding in mass-reared populations suggest a bottleneck followed by a rapid population growth in those commercial populations.

Interestingly, inbreeding was observed in ‘wild’ putative populations. This differential pattern can be explained by the size of the parasitoid populations and the behavior of *A*. *ervi* that tends to reproduce locally [54]. Thus, in natural conditions, where aphid colonies are spatially isolated, mate-finding probability may be low, reducing the effective population size. Conversely, high densities in mass-rearing conditions could favor panmixia within the commercial population. However, the observed inbreeding within wild *A*. *ervi* populations could be the result of a sampling bias. By sampling in several greenhouses within a given region, we could have sampled subdivided populations, artificially increasing F_IS_ values.

In *A*. *ervi* collected in various agroecosystems, a differentiation among specimens from strawberry greenhouses and specimens from other plants was observed. However, this genetic differentiation among crops would be mostly related to the sampling region as no differentiation was further found when considering all specimens from northern regions. Thus, the highest differentiation identified was found between putative populations from Northern regions (‘Center’ and ‘West’) and putative populations from Southern regions of France (‘Southeast’ and ‘Southwest’). This may be the result of isolation by distance, as found by Hufbauer et al. [50] among populations in North America.

Considering *A*. *ervi* specimens collected in protected strawberry crops, a weak genetic differentiation correlated with the host species was observed. This was supported by DAPC only, where individuals emerging from the three major aphid species colonizing strawberry crops clustered together with some overlap. Further sampling of individuals from each aphid species in the same location is needed to disentangle the effect of the aphid host from the sampling location. Host-associated genetic differentiation might be common in parasitoids [55] but was not demonstrated in *A*. *ervi* parasitizing different biotypes of the pea aphid *A*. *pisum* and cereal aphids *Sitobion avenae* and *Rhopalosiphum padi* [30, 56]. In *A*. *ervi*, host-associated differentiation might be prevented by its high phenotypic plasticity [57] and by its relatively high dispersion behavior compared to other Aphidiinae [54].

### Implications for biological control

We need to be cautious not to over-interpret the consequences of the observed structure on *A*. *ervi* efficiency as a biological control agent [58]. Genetic diversity within the released population is not always correlated with the ability of the individuals to regulate the targeted pests [59]. But we can identify potential implications that should be considered for the improvement of biological control.

#### Implications for conservation biological control

In greenhouses where commercial parasitoids were released before sampling, we mostly found ‘wild’ parasitoids. This supports the hypothesis that most parasitism is due to wild individuals rather than released ones, even if greenhouses are relatively closed agroecosystems [13]. Contrary to several other Aphidiinae parasitoids, using molecular markers, *A*. *ervi* was proposed to be a ‘true’ generalist at the scale of an agroecosystem [15]. As no differentiation was observed according to the host plant, we can then expect natural habitats and other crops like Fabaceae crops to be reservoirs of *A*. *ervi* providing control to strawberry greenhouses. Identifying the reservoirs of parasitoids around greenhouses would be a first step to enhance this colonization.

#### Implications for inundative biological control

The mtDNA analysis suggested that low efficiencies in greenhouses cannot be related to the use of a highly differentiated population, with a different host range. However, differences in host ranges are not always visible using COI region [51]. Indeed, *A*. *ervi* is not differentiated from its sister species *A*. *microlophii* although *A*. *microlophii* has a much more restricted host range than *A*. *ervi* [20].

Even though no host-associated genetic differentiation has been observed here, it has been shown that *A*. *ervi* does not equally use its potential host range [15, 57, 60]. This is due to host fidelity through imprinting [57, 60] or genetic factors [61]. So, assuming that the diversity at neutral markers such as microsatellites reflects the diversity at the genome level, the lack of genetic diversity observed in ‘commercial’ *A*. *ervi* may be correlated with a reduction in the range of aphid species efficiently parasitized. It would be necessary to test experimentally the host ranges of populations to verify this assumption. Besides, released parasitoids must overcome the aphid’s defenses, mostly conferred by bacterial symbionts [62]. It has been shown that *A*. *malvae* from strawberry crops infected with the bacterial symbiont *Hamiltonella defensa* were resistant to commercial parasitoids [63]. However, experimental evolution experiments showed that *A*. *ervi* can adapt in a few generations to the protection conferred by *H*. *defensa* in aphids [6]. Assuming this adaptation requires genetic diversity, the lack of genetic variability observed in the ‘commercial’ population might hinder the possibility to overcome the aphids’ resistance. The next step will be to establish the actual relationship between the genetic variability of *A*. *ervi* and its performance on the range of aphids present in strawberry crops (*i*.*e*. multiple aphid species and multiple symbiotic infection statuses). If this relationship is demonstrated, regular checking of genetic diversity in commercial insectaries might help to anticipate supplementation with wild individuals to maintain the potential for adaptation of commercial parasitoids.

## Supporting information

S1 Fig LnPr(X|K) and ΔK plots from STRUCTURE harvester. (PDF)

S2 Fig Genetic structure of ‘wild’ *Aphidius ervi* based on seven microsatellite loci: Inference of population structure using Bayesian clustering with the program STRUCTURE. Coefficient of ancestry is an average on ten runs. (a) K = 2, (b) K = 3.
(PDF)

S1 Table Sequences from GenBank and BOLD used for phylogenetic placement of parasitoids morphologically identified as *Aphidius ervi*. (XLSX)

S2 Table Microsatellite data used for population genetics analyses. (CSV)
