# Supplementary material for: Genetics of wild and mass-reared populations of a generalist aphid parasitoid and improvement of biological control
Source: PLoS One. 2021 Apr 13;16(4):e0249893. doi: 10.1371/journal.pone.0249893 (PMC8043399; doi:10.1371/journal.pone.0249893)
Supplement: S1 Fig — (PDF) [file pone.0249893.s001.pdf]

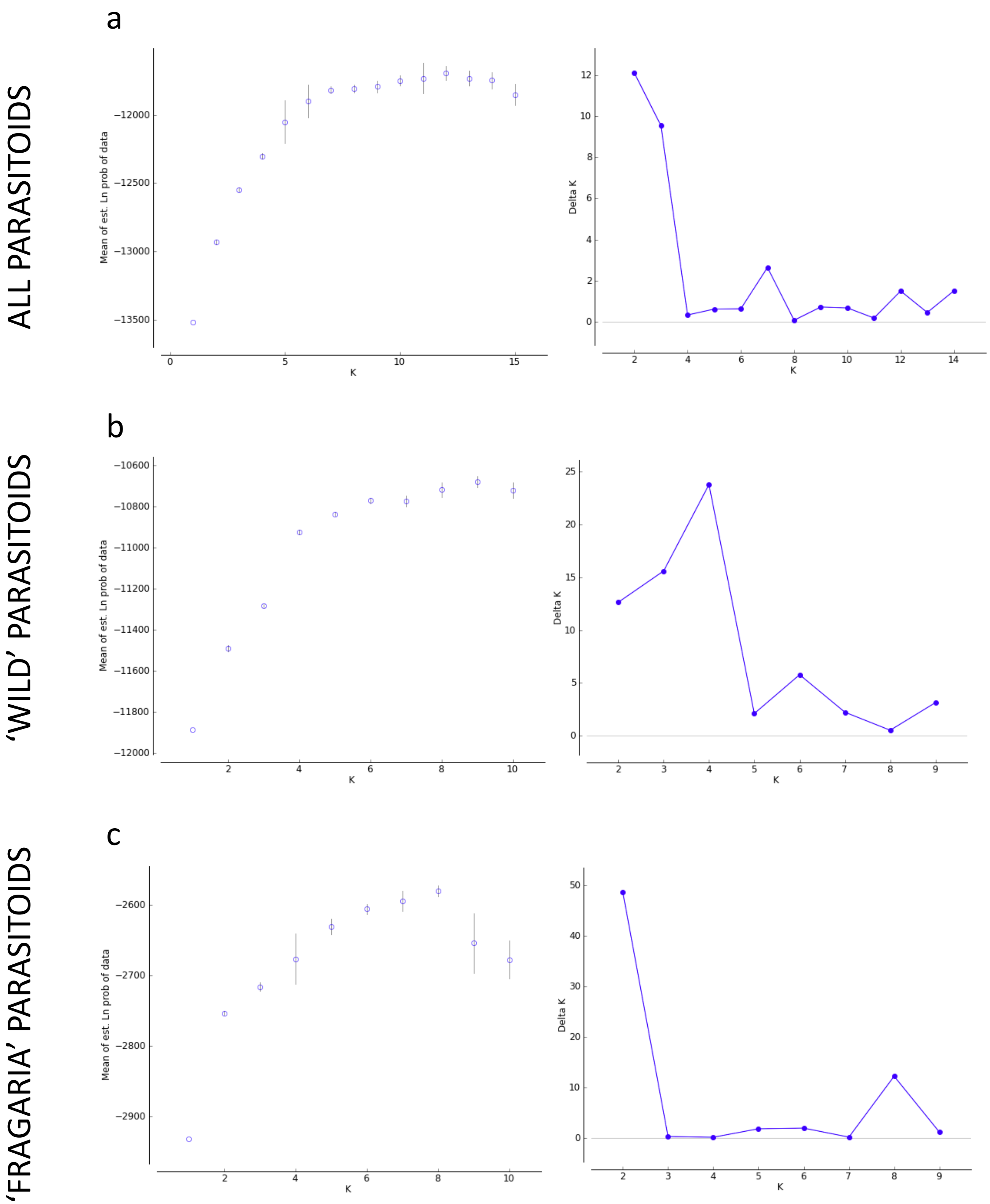

S1 Fig LnPr(X|K) and ΔK plots from STRUCTURE harvester
