# Supplementary material for: Genetics of wild and mass-reared populations of a generalist aphid parasitoid and improvement of biological control
Source: PLoS One. 2021 Apr 13;16(4):e0249893. doi: 10.1371/journal.pone.0249893 (PMC8043399; doi:10.1371/journal.pone.0249893)
Supplement: S2 Fig — Coefficient of ancestry is an average on ten runs. (a) K = 2, (b) K = 3. (PDF) [file pone.0249893.s002.pdf]

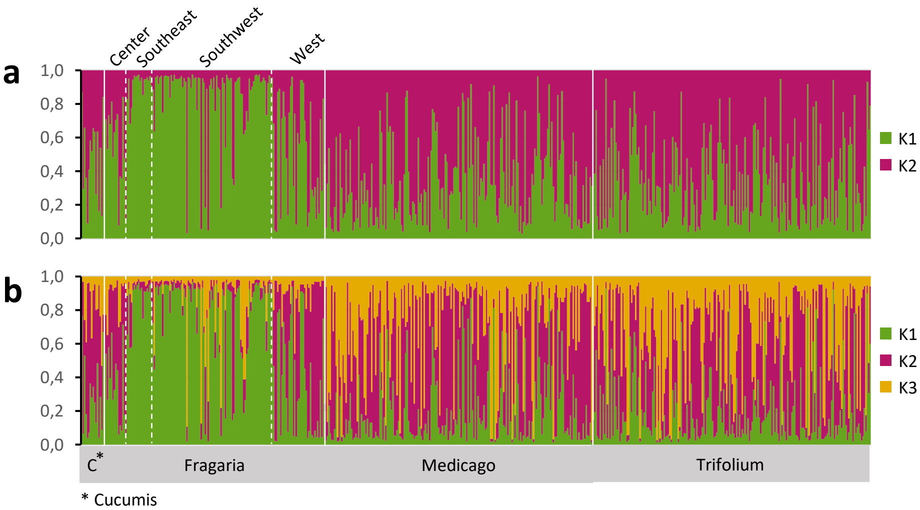

S2 Fig Genetic structure of ‘wild’ *Aphidius ervi* based on seven microsatellite loci: inference of population structure using Bayesian clustering with the program STRUCTURE. Coefficient of ancestry is an average on ten runs. (a) K = 2, (b) K=3.
